# Mathematics Anxiety: An Intergenerational Approach

**DOI:** 10.3389/fpsyg.2020.01648

**Published:** 2020-07-21

**Authors:** Kiran Vanbinst, Elien Bellon, Ann Dowker

**Affiliations:** ^1^Parenting and Special Education Research Unit, Faculty of Psychology and Educational Sciences, KU Leuven, Leuven, Belgium; ^2^Department of Experimental Psychology, University of Oxford, Oxford, United Kingdom

**Keywords:** mathematics anxiety, arithmetic, intergenerational correlations, biological mother and father, primary school children

## Abstract

This study investigated mathematics anxiety from an intergenerational perspective, by investigating data on 172 primary-school children and both their biological parents. This family dataset (*n* = 516) allowed us to not only replicate previous findings per generation but also, importantly, explore intergenerational correlations. We found a significant negative association between sixth graders’ arithmetical performance and their mathematics anxiety. Gender differences occurred in each generation: females were more anxious than males about mathematics. Interestingly, these gender differences were not found in actual arithmetical performance. Analyses of our intergenerational data revealed that children’s mathematics anxiety was significantly associated with their mothers’ mathematics anxiety and both their mothers’ and fathers’ educational level. Regression analyses revealed that the significance level of mothers’ mathematics anxiety became borderline when considering mathematics anxiety and educational level of both parents simultaneously. Interestingly, mathematics anxiety as well as educational level of both biological parents was associated, suggesting that mathematics anxiety results from a complex entanglement of nature and nurture. Current intergenerational data suggest a complex familial basis of mathematics anxiety and indicate that the investigation of parental levels of education and mathematics anxiety contributes to the understanding of individual differences in children’s arithmetic performance.

## Introduction

It is generally considered that parents have an important influence on children’s behavior, attitudes, performance, etc., and that this influence is shown in various domains of children’s lives. To date, however, it is still unclear how parental attitudes toward mathematics relate to children’s performance in, and attitudes to, mathematics and whether gender differences may play a role in such relationships. Some studies suggest that parental attitudes to mathematics may be associated with children’s performance in, and attitudes to, mathematics (Gunderson et al., 2012; Casad et al., 2015; Maloney et al., 2015). This appears, however, to be moderated by the extent to which parents are actually involved in their children’s mathematics learning.

For example, Maloney et al. (2015) found that high mathematics anxiety in parents was only associated with high mathematics anxiety in their children if the parents also had high involvement in their children’s mathematics homework. Presumably, these increased opportunities for parents to model their own fear and dislike for mathematics are transferred to the children, as well as their excessive perfectionist and/or anxiety-provoking approaches that might extend their fear of failure to their children. Notwithstanding this indirect link, the direct association between children’s and parents’ mathematics anxiety was not investigated in that study. Interestingly, Casad et al. (2015) investigated the direct association between children and parents in terms of mathematics anxiety. These researchers asked teenagers as well as one of their parents to fill in a short questionnaire for mathematics anxiety and found an intergenerational association. This transmission across generations turned out to be particularly striking within gender groups, especially between mothers and daughters. However, only one of the parents was included in this study. It would be interesting to check for the level of mathematics anxiety of the other parent, as this might influence the findings. It is important to highlight that the majority of studies investigating children and parents jointly have drawn conclusions based on data obtained through one parent, usually the mother. Some studies also refer to parents when considering children’s main caregivers, who may also be grandparents, nannies, etc. This approach automatically takes as a starting point that transgenerational transmission of mathematics anxiety must exclusively be an environmental transmission, but this is not necessarily the case: Wang et al. (2014) found that 40% of variance in mathematics anxiety is genetically driven, whether directly or as a result of genetic contributions to mathematical ability and to general anxiety, both of which are significant determinants of mathematics anxiety.

Another issue that may influence the relationship between parents’ and children’s mathematics anxiety is that mothers and fathers—even when both are present and participate in their children’s upbringing—may have different types and levels of influence on their children’s attitudes and performance in mathematics. One might, for example, expect that there might be a higher correlation between mothers’ and children’s attitudes to mathematics than between fathers’ and children’s attitudes to mathematics because the majority of children spend more time with their mothers than with their fathers (OECD, 2017). An additional factor that may differentiate associations between mothers’ and fathers’ attitudes to mathematics and those of their children is that on average women are likely to have more negative attitudes to mathematics and, in particular, to experience greater mathematics anxiety than men (Hembree, 1990; Else-Quest et al., 2010; Devine et al., 2012; Hill et al., 2016). There are a few studies that examine differential links between mothers’ and fathers’ attitudes to mathematics and those of their children. Del Rio et al. (2017) carried out a study in Chile where they found that parental levels of mathematics anxiety had little direct association with children’s mathematics performance but appeared to have an indirect effect through influencing the extent of formal home numeracy practices. Parents, who reported lower levels of mathematical anxiety, reported engaging more frequently in advanced numeracy practice with their children. However, only mothers’ home numeracy practices predicted their children’s mathematics performance; fathers’ practices did not.

Studies on the relation between parental mathematics anxiety and child mathematics anxiety are limited, but numerous studies explored other aspects of math attitudes, such as math value, self-efficacy, and gender stereotypes (e.g., Jacob and Bleeker, 2004; Gunderson et al., 2012; Simpkins et al., 2015). Differences between mothers’ and fathers’ attitudes to mathematics have sometimes been studied specifically with regard to gender stereotypes that boys are better at mathematics than girls, rather than with regard to mathematics anxiety as such. Relationships between gender stereotypes and mathematics anxiety would seem particularly likely to occur in females, especially given the influences of “stereotype threat,” i.e., a situational predicament felt in situations where one can be judged by, or treated in terms of, or self-fulfill negative stereotypes about one’s group (Spencer et al., 1999), but the relationship may be somewhat indirect. For example, Del Rio et al. (2019) found in a Chilean sample that both fathers and mothers showed gender stereotypes regarding mathematics on both implicit and explicit measures; that fathers did so to a greater extent than mothers; and that fathers associated themselves with mathematics more than mothers did. Importantly, their gender stereotypes appeared to predict their children’s gender stereotypes and self-concepts, even by the age of 5. Five-year-old boys (but not girls) already showed the stereotype, in implicit measures, that mathematics is for boys. Mothers’ mathematics self-concepts were positively related to their daughters’ mathematics self-concepts, whereas fathers’ mathematical self-concepts were negatively related to their daughters’ mathematics self-concepts. These are striking results that need careful framing of (potential) gender differences and their origins.

Somewhat similarly, Jacobs (1991; also see Bleeker and Jacobs, 2004) found that gender stereotypes about mathematics in fathers were associated with higher self-rating and higher actual performance in mathematics by their sons, but lower self-rating and lower actual performance by their daughters. Gender stereotypes about mathematics in mothers were associated with lower self-rating and lower performance in mathematics by both sons and daughters. Path analyses suggested that parents’ stereotypes interacted with the gender of their child and thereby directly influenced the parents’ beliefs about the child’s abilities. These beliefs in turn influenced their children’s self-perceptions, and both the parents’ stereotypes and the children’s self-perceptions appeared to influence the children’s performance. Differences between fathers’ and mothers’ influences may indicate that fathers’ gender-based expectations about their children were self-fulfilling. In the case of mothers, gender stereotyping may have been associated with their having a negative personal attitude to mathematics, which may have been transmitted to children of both genders.

Tomasetto et al. (2015) also found that associations between parents’ gender stereotyping and their children’s gender stereotypes and self-concepts differed depending both on the gender of the parent having the stereotypes and on the gender of the child. They studied a group of Italian 6-year-olds and their parents and found that both fathers’ and mothers’ evaluations of their children’s mathematical ability were associated with their children’s self-ratings of their own mathematical ability. However, mothers’, but not fathers’, gender stereotyping with regard to mathematics was associated with their daughters’ self-ratings: girls, whose mothers had strong gender stereotypes in favor of boys, gave lower ratings of their own performance. Boys’ self-rating was not associated with gender stereotyping by either parent. The literature reviewed above illustrates some differences in attitudes and emotions across gender, which may be misinterpreted as indicating fundamental inherent differences between genders. However, we emphasize that these findings are not a final point of conclusion but must encourage researchers to study the origin of these gender differences with the intention to tackle them.

The mechanisms at play in the context of gender stereotypes might also play a role in the context of intergenerational associations for mathematics anxiety. The current study will focus on these intergenerational associations regarding mathematics anxiety. One important issue that seems not to have been examined is the extent to which fathers’ and mothers’ mathematics anxiety might be correlated. One might expect such a correlation, due to assortative mating and perhaps also to mutual communication of attitudes toward mathematics leading to mutual influences on each other’s mathematics anxiety. If we are to understand the respective associations between fathers’ and mothers’ attitudes and their children’s attitudes and performance, it is important to be able to take any such correlations into account. As mathematics anxiety is often found to be associated with avoidance of mathematical content in high school and college (Hembree, 1990; Ashcraft et al., 1998) and with a tendency to choose less mathematical focused careers later on (Chipman et al., 1992), the current study additionally included parental educational level.

Against this background, four aims were addressed in the current study. The first aim of this study was to replicate the previously reported association between mathematics anxiety and individual differences in children’s arithmetic ability. Based on several meta-analyses, we expected a moderate, negative relationship between mathematics anxiety and arithmetical performance (e.g., Hembree, 1990; Namkung et al., 2019). The second aim was to check for gender differences, as it has often been found that girls experience higher levels of math anxiety than boys (e.g., Hembree, 1990; Devine et al., 2012; Hill et al., 2016). Gender differences in mathematics anxiety are not only important in themselves but could affect parent–child correlations with regard to mathematics anxiety: the topic to be discussed below. In accordance with existing literature, we predicted that girls would show greater mathematics anxiety than boys but that this would not be associated with differences in actual mathematics performance, as previous studies rarely find these with primary age children and only inconsistently with secondary age children (Dowker et al., 2016).

The third aim was to explore whether children’s mathematics anxiety would be associated with their parents’ mathematics anxiety. Only biological parents were included in this study, and not also adoptive or step-parents, in order to ensure that the potential genetic influences of parents and children were similar for all children in the sample. Wang et al. (2014) found that 40% of variance in mathematics anxiety can be explained by genetic factors. Further, we decided to include both parents, because this has, as far as we know, never been studied previously with regard to mathematics anxiety, though as indicated above, the predictive roles of mothers’ and father’s attitudes have been compared with regard to gender stereotyping and have shown different results depending on gender of the parent. Between-parent correlations might partially explain these results. It is also important to take account of the possibility that any differences found between fathers’ and mothers’ predictive roles in children’s attitudes and performance might be due not to gender effects but to the fact that one parent often spends more time with the children (OECD, 2017). Therefore, in the present study, we sought to investigate which of the parents had a greater caring role for each child and whether this results in differences in the correlations between the parents’ mathematics anxiety and their children’s anxiety and performance. We predicted that maternal as well as paternal mathematics anxiety would be associated with their children’s mathematics anxiety and their performance and that the anxiety level of the parent with the greatest caring role would be the strongest determinant. In addition, we aimed to investigate whether the strength of these associations between parents and children would differ depending on whether the child and parent share the same gender.

The fourth aim of this study was to explore associations between mothers’ and fathers’ in mathematics anxiety and educational level. It was predicted that mothers would show greater mathematics anxiety than fathers. It was also predicted that fathers’ mathematics anxiety would be correlated with mothers’ mathematics anxiety.

## Materials and Methods

### Participants

This study was conducted in Belgium and based on a total sample of 516 participants, composed of 172 children (102 girls, 70 boys) and their biological mothers (*n* = 172) and biological fathers (*n* = 172). The children were in sixth grade, and their average age was 11 years 3 months (*SD* = 5 months, minimum = 11 years, maximum = 12 years). The mothers and fathers had an average age of 41 years 7 months (*SD* = 4 years, 6 months, minimum = 33 years, maximum = 54) and 44 years and 1 month (*SD* = 5 years, minimum = 33 years, maximum = 65), respectively. From the 516 participants, 16 (15 children and one father) were diagnosed with a developmental disorder (dyslexia, dyscalculia, attention deficit hyperactivity disorder, or autism) and 17 (three children, 14 parents) did not have the Belgian nationality. Parents of all children received an information sheet on the study and provided written informed consent for the participation of the family (child and both parents). The children all gave verbal agreement before participating. Initially, 618 families were invited to participate in the study, and approximately one-third of the families responded to the invitation. The analyses were conducted on 172 families, by applying the following criteria: (1) Both parents gave informed consent for family participation; (2) tasks and questionnaires were completed by child and both parents; (3) none of the children repeated or skipped a grade; and (4) all children were taught at school in Dutch from the age of 2 years and 6 months, and both parents mastered the Dutch language sufficiently to complete the questionnaire without difficulties. The study and consent procedures were approved by the Social and Societal Ethics Committee of the University of Leuven, Belgium (G-2016 03 533).

### Materials

#### Children

##### Arithmetic

Children’s arithmetic competence was assessed with an extended version (van Bergen et al., 2012) of the Tempo Test Arithmetic (De Vos, 1992). The addition subtest as well as the subtraction subtest of this timed achievement test was presented. The original subtests involved 40 problems of increasing difficulty, and to avoid ceiling effects in high math achievers, both these subtests were extended with 20 additional additions or subtractions. Children had to solve as many single-digit and multi-digit additions as possible within 1 min. Afterwards, children completed the subtraction subtest in the same way. The score on this test is the number of correctly solved problems on each subtest within the time limit per subtest (maximum = 120). This test combines speed and accuracy into one index score. The test manual reports that the reliability estimate of this test is 0.92.

##### Mathematics anxiety

We used a short version of the Mathematics Anxiety Rating Scale for Adolescents (MARS-A; Suinn and Edwards, 1982) to assess mathematics anxiety of children in primary education. Bosmans and De Smedt (2015) translated and reduced the original questionnaire to 15 items (Cronbach α = 0.88; Cronbach α in current sample = 0.873). Each item consists of a mathematical situation that children often experience (i.e., “How nervous are you when you are called during math class”). Children were asked to indicate on a five-point Likert scale, ranging from not at all (=1) to very anxious (=5), how anxious they were in each specific situation. The score on this test is the average score on the Likert scale (maximum = 5); the higher the score, the more math anxious the child is.

#### Parents

##### Mathematics anxiety

The Mathematics Anxiety Rating Scale Revised (MARS-R; Plake and Parker, 1982) was used to evaluate the parents’ mathematics anxiety of the parents. This revised and shortened questionnaire from 1982 consists of 25 items which were selected from the original questionnaire, i.e., Mathematics Anxiety Rating Scale (Richardson and Suinn, 1972), consisting of 98 items. By using a five-point Likert scale, all parents were asked to indicate for each item how anxious they would be in such a situation (1 = not at all anxious, 5 = very anxious). They also had the option to respond, “not applicable.” The score on this test is the average score on the Likert scale (maximum = 5), with a high score suggesting a high degree of mathematics anxiety (Cronbach α mothers = 0.962; Cronbach α fathers = 0.958).

##### Educational level

All the parents involved in this study were asked to indicate the highest degree they obtained. They could choose from the following response options: (1) no degree, (2) primary education, (3) secondary education, and (4) higher education.

##### Caregiving

All parents and children received the following question: “*Who spends most time with the child?*” providing an indication of who the main caregiver is. According to their own estimation, they could respond mother (=1), equal (=2), or father (=3). By calculating the sum of the responses of each family member, we created a variable that indicates the distribution of care between parents. If the child, mother, and father in a family indicated the mother as the main caregiver, this would result in a total score of 3, versus if all family members designated the father as the main caregiver, this would result in a total score of 9. A score of 6 would indicate that the care of the child was equally distributed between both parents.

### Procedure

This study was conducted with the help of 20 primary schools who invited 618 families to participate to this study. Only the families from which both parents agreed to participate to the study and who both completed the questionnaires without missing data were selected for the current study. Eventually, 172 entire families (mother, father, and sixth grader of each family) participated to the study. All 172 sixth graders were tested in their classroom by completing a group-based arithmetic task. They also filled in an age-appropriate mathematics anxiety questionnaire. All 344 parents completed a mathematics anxiety questionnaire adapted to the living environment of adults. They could fill in the questionnaire at home whenever they wished and gave it back in a sealed envelope. We additionally requested background information such as educational level, but anonymity was guaranteed as we did not ask for identification data.

## Results

The results of this study are structured and reported with regard to the four study aims.

### Aim 1: Replicating Previous Findings as to the Relationship Between Anxiety and Performance

The first aim of this study was to investigate the association between children’s mathematics anxiety and their arithmetic ability. The descriptive statistics of the measures of the children are presented in Table 1, together with parental measures.

**TABLE 1:** Descriptive statistics (*n* = 516).

	*n*	*M*	*SD*	Min	Max	Theoretical max
**Children**						
Arithmetic	172	55.51	8.08	32	86	120
Mathematics anxiety	172	1.68	0.48	1	3.4	5
**Parents**						
Mothers’ mathematics anxiety	172	2.15	0.81	1	4.40	5
Fathers’ mathematics anxiety	172	1.69	0.64	1	4.92	5

*Min, minimum; Max, maximum.*

As illustrated in Table 2, we found a significant association between children’s arithmetic ability and their mathematics anxiety, with a Bayes factor indicating strong evidence in favor of an association.

**TABLE 2:** Correlations between variables of interest (*n* = 516).

		1	2	3	4	5	6
**Children**							
1. Arithmetic	*r*	–					
	*p*	–					
	*BF10*	–					
2. Mathematics	*r*	−0.307	–				
anxiety	*p*	<0.001					
	*BF10*	>100	–				
**Mothers**							
3. Mathematics	*r*	0.105	0.180	–			
anxiety	*p*	0.171	0.018				
	*BF10*	0.242	1.504	–			
4. Educational	*r*	0.055	−0.176	−0.240	–		
level	*p*	0.475	0.021	0.002			
	*BF10*	0.123	1.333	13.810	–		
**Fathers**							
5. Mathematics	*r*	−0.001	0.132	0.193	−0.062	–	
anxiety	*p*	0.987	0.083	0.011	0.417		
	*BF10*	0.095	0.422	2.354	0.132	–	
6. Educational	*r*	0.095	−0.178	−0.052	0.510	−0.028	–
level	*p*	0.221	0.021	0.500	<0.001	0.713	
	*BF10*	0.203	1.367	0.121	>100	0.103	–

*Pearson correlation coefficients (*r*) as well as Bayes factors (*BF*_10_) were calculated to examine correlations between all variables via the JASP 0.8.4.0 software (JASP Team, 2018).*

### Aim 2: To Investigate Gender Differences in Children’s Mathematics Anxiety and Mathematical Performance

When looking for gender differences, we found that in levels of mathematics anxiety, girls (*M* = 1.771, *SD* = 1.506) were more anxious about mathematics-related situations than boys (*M* = 1.536, *SD* = 1.412), *t*(170) = −3.220, *p* = 0.002. These gender differences were not observed in actual mathematics performance, *t*(170) = 1.703, *p* = 0.090 (girls: *M* = 54.65, *SD* = 7.971; boys: *M* = 56.77, *SD* = 8.131).

### Aim 3: To Investigate Intergenerational Associations

The third aim of the current study was to explore associations between children’s math anxiety and math anxiety of both parents (see Table 2). Interestingly, children’s mathematics anxiety was significantly correlated with maternal mathematics anxiety, though the Bayes factor indicated there was only a trend toward such an association. Children’s mathematics anxiety was marginally significantly associated with paternal mathematics anxiety (*p* = 0.083) and the Bayes factor indicated some support for the null hypothesis. We additionally used the Williams–Steiger test (Lee and Preacher, 2013) to investigate whether the strength of the association between mathematics anxiety of parents and children, differed in terms of the parent’s and child’s gender. The strength of the association between girls and their mothers did not differ significantly from the strength of the association between these girls and their fathers, *z* = −0.664, *p* = 0.253. Similarly, no gender differences were found for the strength of the association between mathematics anxiety of boys and their mothers versus fathers, *z* = −0.239, *p* = 0.405. For children’s arithmetic ability, no correlations were observed with parental mathematics anxiety, and the Bayes factors indicated evidence in favor of the null hypotheses.

The parents’ educational level was also examined. Among the mothers, 1% had no educational qualifications, 2% completed primary education, 31% completed secondary education, and 66% obtained a higher education degree. In the group of fathers, 2% had no educational qualifications, 4% completed primary education, 37% completed secondary education, and 57% obtained a higher education degree. The mathematics anxiety of the mothers was associated with their own educational level (with a Bayes factor indicating strong evidence for this association), reflecting that higher educational level was associated with lower mathematics anxiety. Such an association was not found for the fathers.

Children’s mathematics anxiety was significantly correlated with the educational level of both parents, although Bayes factors showed less clear significance. The Williams–Steiger test (Lee and Preacher, 2013) was used to investigate gender differences. The strength of the association between girls’ mathematics anxiety and their mothers’ educational level did not differ significantly from the strength of the association between these girls’ mathematics anxiety and their fathers’ educational level (*z* = −0.406, *p* = 0.341). Similarly, the strength of the association between boys’ mathematics anxiety and their fathers’ versus mothers’ educational level did not differ significantly, *z* = −0.064, *p* = 0.474).

A regression analysis was used to investigate whether children’s mathematics anxiety was associated with their mothers’ and fathers’ mathematics anxiety and educational level. The model presented in Table 3 illustrates that when all parental variables were simultaneously entered in the regression model, maternal mathematics anxiety turned out to be the only marginally significant determinant of children’s mathematics anxiety. The Bayes factor inclusion provided moderate evidence for this. In contrast, maternal educational level and paternal mathematics anxiety as well as educational level were not significantly associated with children’s mathematics anxiety.

**TABLE 3:** Regression analysis predicting children’s mathematics anxiety based on parental mathematics anxiety and educational level (*n* = 516).

Children’s mathematics anxiety	*B*	*t*	*p*	*BF*_*inclusion*_
**Mothers**				
Mathematics anxiety	0.154	1.951	0.053	2.420
Educational level	−0.079	0.885	0.377	0.789
**Fathers**				
Mathematics anxiety	0.089	1.163	0.247	0.561
Educational level	−0.127	1.453	0.148	1.285

**F*(4,168) = 3.520, *p* = 0.009, *R*^2^ = 0.079.*

To test the hypothesis that care moderates the relationship between children’s mathematics anxiety and parental mathematics anxiety, children, mothers, and fathers were asked to indicate with whom they estimated the child was spending most of his/her time: the mother, the father, or both equally. In the group of children, 58% responded that they spent an equal amount of time with each parent, 38% responded that they spent most time with their mother, and 4% responded that they mostly spent time with their father. The responses of the children correlated with the responses of the mothers (*r* = 0.384, *p* < 0.001) and the fathers (*r* = 0.420, *p* = 0.001). The mothers’ and fathers’ responses correlated particularly strongly with one another c (*r* = 0.744, *p* < 0.001). In most cases, both parents indicated either that the children spent most of their time with the mother (52% according to both mothers and fathers) or that they spent equal amounts of time with the mother and the father (47% according to mothers, 44% according to fathers). Only a small minority of the parents indicated that the child spent most of their time with the father (1% according to the mother, 4% according to the father).

For subsequent analyses, we calculated the sum of the responses of children, mothers, and fathers, as an indication of the distribution of care between parents (*M* = 4.657, *SD* = 1.357). A total score of 3 indicates that all three family members considered the mother as the main caregiver (30%), a total score of 9 indicates that the father was designated as the main caregiver (0.5%), and a total score of 6 refers to an equal division (30%). If the child, mother, and father responded differently, in-between values were created, i.e., score of 4 (21%), score of 5 (14%), score of 7 (4%), and score of 8 (0.5%).

A moderation analysis was conducted to examine whether the caregiving score affected the association between mothers’ mathematics anxiety and children’s mathematics anxiety. This analysis was only conducted for mother and child, as the association between fathers’ mathematics anxiety and children’s mathematics anxiety was not significant. The moderation analysis was performed using the PROCESS computational tool for SPSS (Hayes, 2018). Table 4 presents this overall significant model, *R*^2^ = 0.054, *F*(3,168) = 3.19, *p* = 0.025. The interaction term between mothers’ mathematics anxiety and care was a significant determinant of children’s mathematics anxiety. Simple-slope analysis showed that the relationship between the mathematics anxiety of the children and the mathematics anxiety of the dominant caregiver was significant (*b* = 0.13, 95% CI [0.05–0.22], *t* = 3.00, *p* = 0.003), while this was not the case for children for which care is more equally distributed between parents (*p* > 0.05).

**TABLE 4:** Linear model of determinants of children’s mathematics anxiety.

	*b*	*SE b*	*t*	*p*
Constant	3.71	3.32	1.12	0.26
Mother’s mathematics anxiety	0.26	0.10	2.50	0.01
Care	1.01	0.71	1.41	0.16
Mother’s mathematics anxiety × care	−0.04	0.02	−1.94	0.05

**R*^2^ = 0.054.*

### Aim 4: Parental Associations?

Correlations between parents were significant, indicating associations between mothers’ and fathers’ mathematics anxiety (with a Bayes factor indicating moderate evidence for the association) as well as between mothers’ and fathers’ educational level (with a Bayes factor indicating strong evidence in favor of this association). Mathematics anxiety measurements of the parents indicate that mothers were significantly more anxious in math-related situations than fathers, *t*(171) = 6.496, *p* < 0.001.

## Discussion

Consistent with existing studies, our first prediction was supported as there was a highly significant negative correlation between children’s arithmetical performance and their anxiety. This is in line with many previous studies (Hembree, 1990; Ashcraft and Faust, 1994; Ma and Kishor, 1997; Ho et al., 2000; Carey et al., 2016; Foley et al., 2017; Pitsia et al., 2017; Namkung et al., 2019). It is true that findings with primary-school children (Krinzinger et al., 2009; Dowker et al., 2012) often show relatively little correlation between performance and mathematics anxiety, especially with regard to what Wigfield and Meece (1988) termed the “worry” (performance anxiety) dimension. There may be earlier development of such a correlation with regard to the “emotionality” dimension of fear and anxiety elicited by the very presence of mathematical stimuli (Wigfield and Meece, 1988; Vukovic et al., 2013; Sorvo et al., 2017). However, usually by the age of 11, children have come to show signs of a correlation between mathematics anxiety and mathematical performance (Hembree, 1990; Ma and Kishor, 1997; Hill et al., 2016). This was indeed found in our group of sixth graders.

Our second prediction was also supported: in this sixth-grade group, girls were more anxious than boys about mathematics. This replication is in line with results by a number of researchers with regard to secondary-school pupils (Devine et al., 2012; Wu et al., 2012; Harari et al., 2013; Dowker et al., 2016) and adults (Else-Quest et al., 2010; Ferguson et al., 2015). Results are a little less consistent with primary-school children, but gender differences in mathematics anxiety are certainly sometimes found (Hill et al., 2016). It should be emphasized that this does not reflect an actual consistent superiority by males in actual mathematical performance. The present study showed no gender difference in mathematics test performance. In the past, there was a tendency for secondary-school pupils, though often not primary-school pupils, to show a gender difference in mathematics performance in favor of boys. This is likely to be largely due to cultural factors, as the gender gap varies a lot between countries, and is decreasing over time, as shown by the successive TIMSS international studies of pupils’ mathematical performance (Mullis et al., 2015, 2016a,b). When international surveys combine data from many countries, there is still some advantage in secondary school in favor of boys, but this is small and is not found in all countries, and a few countries such as Jordan and Malaysia even show an advantage for girls. Interestingly, even in Malaysia, where girls have for some time done better than boys in mathematics tests, boys still tend to rate themselves higher in mathematics than girls do (Ismail and Awang, 2008). This illustrates the complex interaction between perceptions (by oneself and others) and actual educational achievements.

The third prediction (i.e., children’s mathematics anxiety is related to their parents’ mathematics anxiety) was also supported, though with qualifications. Children’s mathematics anxiety was significantly associated with their mothers’ mathematics anxiety, and interestingly with their mothers’ as well as fathers’ educational level. In the regression, we predicted children’s mathematics anxiety based on the mathematics anxiety as well as educational level of both parents. We found that when these four parental measures were considered simultaneously, only one determinant of children’s mathematics anxiety remained marginally significant, namely, mothers’ mathematics anxiety. In contrast with some previous findings (Jacobs, 1991; Del Rio et al., 2019), there appeared to be no difference between boys and girls regarding associations with maternal versus paternal mathematics anxiety and their own mathematics anxiety. Consistent with many previous findings of gender differences in mathematics anxiety (Hembree, 1990; Else-Quest et al., 2010; Devine et al., 2012; Ferguson et al., 2015; Hill et al., 2016), mothers showed higher levels of mathematics anxiety than fathers did. Our results should be interpreted in this context, especially given the between-parent correlations observed for mathematics anxiety as well as educational level. In contrast to the group of mothers, mathematics anxiety and educational level were not significantly associated in the group of fathers. Concretely, lower levels of mathematics anxiety co-occur with higher levels of education, or vice versa, and higher levels of mathematics anxiety go together with lower education levels. We argue below that the existence of mathematics anxiety should be taken seriously, as mathematics anxiety might be a threat to the general level of education obtained (by girls). If future studies provide evidence for the observation that the general academic achievement of boys is less affected by mathematics anxiety compared to girls, than we have a clear argument for the need to urgently tackle math anxiety, especially in the female population.

Care moderated the relationship between children’s mathematics anxiety and their dominant caregivers’ mathematics anxiety: the level of the correlation between children’s and mothers’ mathematics anxiety depended on whether their mother was their main carer. Thus, it is likely that the greater association between children’s mathematics anxiety and their mothers’ than their fathers’ mathematics anxiety was due to mothers’ greater involvement in their children’s everyday lives. Tackling mathematics anxiety from an early age on might be crucial for breaking through the mutually reinforcing association between mathematics anxiety and arithmetical performance, with a potential transgenerational transmission.

Although the association between mathematics anxiety of children and their mothers was higher than the association between mathematics anxiety of children and their fathers, we want to highlight that the following findings should be interpreted with care (see also Jackson and Mannix, 2004; Ladd-Taylor, 2004). Based on these data, responsibility cannot and should not be placed with the mother (see Courcy and des Rivières, 2017, for a similar rationale in the context of autism spectrum disorders). First, it should be noted that variance in mathematics anxiety was higher in mothers than in fathers, and as such, significant correlations may be more easily observed. Second, correlations between parents were significant at the level of their mathematics anxiety but also their educational level. We wonder how our findings would look like if we tested highly anxious fathers who also play a bigger role in the everyday lives of their children compared to mothers. Would the same pattern of findings be found? With this in mind, we believe that the current intergenerational findings highlight the urgency of tackling math anxiety, especially in the female population.

It is of interest that mothers’ level of mathematics anxiety was associated with their own educational level, while this was not the case for fathers. This may mean that mathematics anxiety (or possibly general academic anxiety) influences girls’ educational progress more than it does for boys and/or that girls’ educational experience influences their mathematics anxiety more than it does for boys. If the first is the case, it could mean that girls’ mathematics anxiety has a more potentially deleterious effect on their subsequent educational progression than is the case for boys. This would need more investigation, especially as the parents by definition had received their education a generation ago, and educational and social changes might result in different associations nowadays. At any rate, the results indicate the importance of looking not only at gender differences in actual levels of mathematics anxiety but also at gender differences in the extent and manner in which they influence educational outcomes.

The current study showed, as predicted, that children’s mathematics anxiety was associated with their arithmetic performance. There was evidence for an association between mathematics anxiety of children and their mothers, but less evidence for an association between children and their fathers. Interestingly, there was no significant relationship between parental mathematics anxiety and children’s arithmetical performance. This may seem as a puzzling result, but Casad et al. (2015) found the same result in a group of teenagers and their parents. It may be that other factors are mediating and/or moderating these relationships. It must be remembered that although mathematical performance and mathematics anxiety are significantly negatively correlated in most studies, and among the children in this study, other factors are involved in both mathematics anxiety and mathematics performance. For example, Devine et al. (2018) found that, though mathematics anxiety was significantly commoner in children with than without mathematical difficulties, not all children with mathematical difficulties were high in mathematics anxiety, and many children who were performing well in mathematics did show high mathematics anxiety. The interaction between mathematics anxiety versus performance is complex, and this interaction becomes even more difficult to disentangle across generations. Children’s mathematics anxiety was significantly associated with their mothers’ mathematics anxiety, but interestingly it was also significantly associated with the educational level of both parents, illustrating that parental attitudes and general academic achievement are both important for children’s academic progress. It is difficult, on the basis of our current data, to disentangle whether intergenerational transmission of mathematics anxiety results from parental mathematics anxiety versus their general academic ability. The direction of the association between mathematics anxiety and arithmetic remains unresolved, even though some studies seem to provide evidence for the idea that difficulties in arithmetic lead to mathematics anxiety. For example, in a very recent study in slightly younger children (7–9-year-olds) within the same population as the current study, it was found that the predictive association between arithmetic ability and mathematics anxiety was only significant from arithmetic in second grade (7–8-year-olds) to mathematics anxiety in third grade (8–9-year-olds) and not the other way around (Bellon et al., Under Revision). This ongoing chicken-or-egg discussion indirectly suggests bidirectional associations between mathematics anxiety and arithmetic ability across time and even maybe across generations.

It would also be desirable to use a wider variety of mathematics tests, to investigate whether children’s mathematics anxiety is more associated with some aspects of arithmetical performance than with others. In particular, levels of time pressure may influence the effects of anxiety on performance. There is some evidence that mathematics anxiety is higher for timed than untimed tests and also that gender differences in anxiety and sometimes in performance are greater for timed tests (Steinmayr and Spinath, 2019). Differences in extent and type of working memory demands may also influence the effects of mathematics anxiety on performance. For example, DeCaro et al. (2010) found that external pressure, at a level likely to increase anxiety, impaired performance on verbal but not spatial mathematics problems, presumably because anxiety led to a particular overload on verbal working memory.

In particular, the currently studied relationships might be elucidated in future studies by including a measure of arithmetical performance in parents. This would be potentially difficult to implement, especially as parents with high levels of mathematics anxiety are likely to be reluctant to take such a test; but it might give a significant amount of further information about parental determinants of children’s mathematical attitudes and performance. It may be, for example, that parents’ mathematics anxiety determines children’s mathematical performance more when it is combined with weaknesses in the parents’ mathematical performance than when the parents are anxious but are themselves performing well in mathematics.

It may also be that the potential influence of parents’ mathematics anxiety on both their children’s mathematical performance and their children’s mathematics anxiety may depend on the extent to which they are involved in their children’s mathematical activities. As mentioned in Section “Introduction,” Maloney et al. (2015) found that parental mathematics anxiety only had significant effects on children’s mathematics anxiety and performance if they also had significant involvement in their children’s mathematics homework. Although the present study investigated the caring role of each parent, it did not look directly at their involvement in mathematics homework and other aspects of children’s mathematics education. It may be that, although parental mathematics anxiety showed an overall relationship to children’s mathematics anxiety, it was *only* linked to performance if it was combined with heavy involvement in children’s mathematical activities. Future studies should disentangle if in-between parental variability in caregiving time reflects either absolute time spent together or relative time spent on tutoring the child.

The fourth and final prediction—that mothers’ and fathers’ mathematics anxiety would be correlated—was supported, though the correlation was not as strong as for educational level. There could be several reasons for this correlation. Associations between mothers’ and fathers’ mathematics anxiety could be either a direct result of either assortative mating or mutual influence on mathematics anxiety itself or a more indirect result of assortative mating or mutual influence on mathematical ability, general anxiety, or both. Mathematics anxiety is known to be related to both mathematical ability and general anxiety (e.g., Wang et al., 2014). Neither of these was assessed for parents in the present study, so we cannot draw conclusions about the reasons for the interparental associations. They are, in any case, only a focus of the present study insofar as they influence the context for the children’s mathematics anxiety. They mean that any study of associations between parental mathematics anxiety on children’s mathematics, or of differences between fathers and mothers in this respect, must take account of the fact that mothers’ and fathers’ mathematics anxiety levels are not independent. These findings suggest that the entire context of parental mathematics anxiety, including fathers as well as mothers, contributes to mathematics anxiety in children.

The results lead to several avenues for future study. First of all, longitudinal studies would help us to gain greater understanding of parental influences on the development of mathematics anxiety over time and if this may be altered through interventions with both parents. Second, a wider variety of aspects of children’s and parents’ attitudes and emotions toward mathematics should be investigated. These could include active liking for mathematics, self-rating in mathematics, self-efficacy, and reactions to and attributions for success and failure in mathematical activities. It might also be useful to consider the role of motivation (Wang et al., 2018), as mathematics anxiety plays a role in people’s motivation to choose for mathematically embedded educational directions and professions. There could also be an attempt to differentiate the effects of cognitive and affective dimensions of mathematics anxiety (Wigfield and Meece, 1988), especially as previous research has indicated that a relationship between children’s mathematical performance and the affective dimension of anxiety emerges at an earlier age than the relationship between their mathematical performance and the cognitive dimension of anxiety (Sorvo et al., 2017). Third, it would be of interest to investigate families with a wider range of parental educational background. There were rather few parents with very limited education in this study. It is possible that a study including more such parents would indicate stronger associations between both parental educational background and parental mathematics anxiety and their children’s mathematical anxiety and performance. As indicated earlier, this kind of research should be framed in a way that it is not generating mother-blaming.

It should be emphasized that this study was carried out in one particular country, Belgium, and that different results might be obtained elsewhere. The OECD PISA international comparisons indicate that Belgium is a relatively high-scoring country in mathematics performance and that it appears to be close to average among OECD countries for mathematics anxiety (OECD, 2013). A lower scoring country, or one with higher or lower mathematics anxiety, might possibly show a different relationship between parents’ mathematics anxiety and children’s performance. Also, the parents included in the study were mostly relatively highly educated, and this may have partially excluded parents with the highest levels of mathematic anxiety. Future studies should broaden the study of associations between parental mathematics anxiety and children’s performance to include more countries and a wider range of parental education levels.

## Conclusion

In sum, we replicated a well-established finding in the literature that children’s mathematics anxiety was significantly correlated with their arithmetical performance. Girls appeared more anxious about mathematics-related situations than boys did, but these gender differences were not observed in actual mathematics performance. The intergenerational analyses revealed that children’s mathematics anxiety was associated with their parents’ educational level. Mathematics anxiety of children was associated with that of their mothers only in the case of those children whose mother is the dominant caregiver. Interestingly, mathematics anxiety of both biological parents was associated, suggesting that mathematics anxiety involves a complex entanglement of nature and nurture. Our data do not provide evidence for a mother- or father-blaming story but clearly show a complex familial basis for mathematics anxiety.

## Data Availability Statement

The datasets generated for this study are available on request to the corresponding author.

## Ethics Statement

The studies involving human participants were reviewed and approved by the Social and Societal Ethics Committee of the University of Leuven, Belgium (G-2016 03 533). Written informed consent to participate in this study was provided by the participants’ legal guardian/next of kin.

## Author Contributions

KV designed the study, collected the data, and took the lead in writing the manuscript. KV and EB analyzed the data. AD suggested to perform additional analyses. All authors contributed to the interpretation of the results, provided critical feedback, and helped shape and writing the manuscript.

## Conflict of Interest

The authors declare that the research was conducted in the absence of any commercial or financial relationships that could be construed as a potential conflict of interest.
